# Food Consumption and Characteristics Associated in a Brazilian Older Adult Population: A Cluster Analysis

**DOI:** 10.3389/fnut.2021.641263

**Published:** 2021-05-07

**Authors:** Laís M. R. Loureiro, Luciene F. F. Almeida, Carla J. Machado, Milene C. Pessoa, Maria Sônia L. Duarte, Sylvia C. C. Franceschini, Andréia Q. Ribeiro

**Affiliations:** ^1^Department of Nutrition and Health, Universidade Federal de Viçosa, Viçosa, Brazil; ^2^Department of Preventive and Social Medicine, School of Medicine, Universidade Federal de Minas Gerais, Belo Horizonte, Brazil

**Keywords:** food consumption, nutritional epidemiology, eating habits, aging, older adults, cluster analysis

## Abstract

Epidemiological studies support diet as a factor in the prevention and treatment of non-communicable chronic diseases, whose occurrence increases with age due to the poor choices or the adoption of a monotonous diet. The aim of this study was to construct the food consumption profiles of older adults of a Brazilian city to identify the main food groups and eating habits that contribute to these profiles and to estimate its association with socioeconomic characteristics, health and use of health services, lifestyle, and anthropometric indicators. This is a population-based cross-sectional study conducted with a representative sample of 621 community-dwelling older adults (≥60 years) in Viçosa, Minas Gerais, Brazil. The food consumption profile was the dependent variable obtained from a Food Frequency Questionnaire, utilizing the two-step cluster method. The multiple multinomial logistic regression model was used to estimate the independent associations, obtaining the odds ratios and 95% confidence intervals. Three clusters were generated, namely, (1) “unhealthy” (2) “less unhealthy,” and (3) “fairly healthy.” The cluster “unhealthy” was characterized by a regular consumption of beans, fats, fatty/processed meats, and whole milk. The factors independently associated with this cluster were lower education level, lower individual income, history of at least one doctor's appointment in the year preceding this study, and being a former smoker. The cluster “less unhealthy” was characterized by a regular consumption of beans, green vegetables, vegetables and fruits, as well as fats, fatty/processed meats, and whole milk. The factors independently associated with the “less unhealthy” cluster were lower education level and history of at least six doctor's appointments in the prior year. The cluster “fairly healthy” was characterized by the same pattern of “less unhealthy,” except for skim milk and low-fat dairy products. The evidence of the associations indicates the profile of older adults who require greater attention and care related to improved nutrition. The illiterate or semi-literate aged individuals, those with low income, and those who neglect to seek medical advice must be the focus of healthy eating actions and programs.

## Introduction

Maintenance of the independence of older adults as well as their effective participation in society is directly linked to the preservation of their health, which is influenced by various factors, including lifestyle (1). In this context, the epidemiological and review studies offer evidence to support the importance of diet as a factor in the prevention and treatment of various non-communicable chronic diseases like diabetes, dyslipidemia, hypertension, and obesity (2–4), which pose challenges to the health professionals given that their occurrence increases dramatically with advancing age (5). Due to the poor choices or the adoption of a monotonous diet, the aged individuals may experience deficiencies of essential nutrients necessary to maintain health and appropriate disease control (4, 6).

Studies available in the literature also deepen the understanding of the determinants of eating habits in various populations. Within the scope of the older adult population, international studies have demonstrated that higher income, greater education level, and less cognitive impairment are associated with better nutrition (3, 7–9), while diet deficiencies and unhealthy eating habits are associated with male gender, obesity treatment (10), history of myocardial infarction, ischemic heart disease, and heart failure (11). In Brazil, nutritional epidemiology focused on aging is still a relatively new field of study, and only a few studies have investigated the determinants of eating habits of older adults. From these studies, a positive association has been identified between adequate eating habits and female gender, higher education (12–14), advancing age, a greater number of comorbidities (15), non-smoking (13, 14) overweight, and a history of consultation with a nutritionist (13, 14). On the other hand, low diet quality was associated with male gender, age <80 years, lower education, problems in affording food (16), smoking habit, underweight, mouth or teeth problems, and having <4 meals a day (17, 18).

Although there is an increase in the number of studies on food consumption among older adults in Brazil, little is known about this phenomenon in smaller cities. Social, economic, and cultural differences are significant among the Brazilian subpopulations, and the effectiveness of the promotion of healthy eating habits is influenced by these distinctions. In addition, grouping aged individuals according to their eating habits allows the identification of more vulnerable profiles that need more attention from public policies. This approach is still scarce in Brazil.

Thus, the aim of this study is to construct and describe the food consumption profiles of the older adult population of a medium-sized Brazilian city, by identifying the main food groups and eating habits that contribute to these profiles, and to estimate the association of the social and demographic characteristics, health and use of health services, lifestyle, and anthropometric indicators with these profiles.

## Materials and Methods

### Study Design and Participants

This was a population-based, cross-sectional study—part of the project “Health conditions, nutrition, and use of medication by older adults in Viçosa (Minas Gerais): a population-based survey.” Viçosa is a medium-sized Brazilian city located in the region of Zona da Mata in the state of Minas Gerais.

The study population consisted of older adults, aged 60 years or more, who are non-institutionalized residents in the municipality, including rural and urban areas. The target population was 7,980 individuals. The sample size was calculated with a confidence level of 95%, with an estimated prevalence of 50% for different outcomes of interest in the larger project, a tolerated error of 4%, and 20% to cover losses. Based on the calculation, the total number obtained was 670 individuals. There were losses by refusal (*n* = 24, 3.6%) due to death, address not found, and emigration from the city (*n* = 25, 3.7%). Losses did not differ by gender and age group. The participants were selected and interviewed according to the description of Nascimento et al. (19). The final sample consisted of 621 participants.

The interviews were pre-scheduled and conducted in the participants' houses by previously trained nutritionists paired with final-year undergraduate nutrition students. The present study was conducted according to the Declaration of Helsinki. The research project was approved by the Research Ethics Committee of the Federal University of Viçosa (ref. 27/2008). All the participants signed the informed consent form.

### Study Variables

#### Social, Demographic, and Economic Variables, Health Conditions, and Lifestyle

The social and demographic variables of interest included sex (male, female), age range (60–69, 70–79, and 80 years or more), education level (never studied, <8 years of study, 8 years of study or more), income (quartiles), and cohabitation (lived alone, resided with someone else).

The variables of health conditions were self-perception of health (very good/good, regular, poor/very poor), hospital admissions (none, one, or more), and number of doctor's appointments (none, one to five, six or more) in the year preceding the interview as an indicator of healthcare or need for medical care. The functional ability was assessed using the questions regarding the ability to perform 14 basic and instrumental daily life activities, and the answer options included no difficulty, little difficulty, great difficulty, unable to do, and do not do (19). Besides the variable functional ability, the variables of instrumental daily life activities such as difficulty in eating and difficulty in preparing food were considered. The functional ability was defined as inadequate when the participant reported difficulty with the performance of seven or more activities or when they evaluated themselves as unable to perform three or more activities. Otherwise, the functional ability was defined as adequate (20). Polypharmacy, which was considered in this study, is defined as the use of five or more drugs (21). The 15-day recall period was defined in line with the literature to minimize memory bias (21, 22). Regarding the history of chronic disease, the following question was posed: “Even once in your life, has a doctor or any other health professional ever mentioned that you have or have had any of these diseases?” For the analysis, a history of diabetes, high blood pressure, dyslipidemia, and depression were used. The development of the instrument used to obtain information on health conditions was based on validated questionnaires as used by large national health surveys and prospective health studies with older adult populations (23–25).

Regarding their lifestyle, the seniors were questioned about their consumption of alcoholic beverages (yes; no, but used to drink formerly; never drank at all) and smoking (yes; no, but used to smoke formerly; never smoked at all). Besides that, they were questioned about a decrease in food intake due to loss of appetite, digestive problems, and chewing or swallowing difficulties within 3 months prior to the interview (26).

#### Anthropometric Variables

Concerning nutritional status, we calculated the body mass index (kg/m^2^), the waist-to-hip ratio, and the waist-to-height ratio. All these rates were used in the analysis as continuous variables besides waist circumference (27).

#### Food Consumption Variables

Regarding food consumption variables, the data collection instrument was a Food Frequency Questionnaire (FFQ) consisting of a long list of foods and consumption frequency options: once a day, two or more times a day, 2–4 times a week, 5–6 times a week, once a week, twice a week, monthly, and never or rarely. The foods presented in the FFQ were grouped and classified as “healthy” and “unhealthy” eating indicators. Food groups classified as healthy eating indicators included beans, green vegetables, other vegetables, fruits, skim milk, and low-fat dairy products. Food groups classified as unhealthy eating indicators were whole milk, soda, processed foods in general, sweets, fatty /processed meats, and fats (saturated and trans-fat) (Table 1). According to the methodology of a national study in Brazil, entitled Protective and Risk Factors for Chronic Diseases by Telephone Survey (VIGITEL) of the year 2016, the consumption of each group was considered “regular” when some foods of the group were consumed at least five times a week and was considered “non-regular” when it was consumed up to four times a week. The exceptions were the consumption of whole milk, fats, and fatty/processed meats, which were considered unhealthy eating indicators with a regular consumption even when they were consumed once a week (28).

**Table 1:** Classification of foods in groups of healthy and unhealthy eating indicators.

**Food Groups**		**Foods**
Healthy Eating Indicators	Beans^a^ and Green vegetables^a^	Black, brown, and red beans
	Other vegetables^a^	
	Fruits^a^	
	Skim milk and low-fat dairy products^a^	Skim milk
		White cheese (Minas cheese^d^)
		Ricotta
Unhealthy eating indicators	Whole milk^b^	
	Soda^c^	
	Processed foods in general^c^	Cracker
		Cornstarch cookie
		Pound cake
		Lasagna
		Fried “pastel”^e^
		Pizza
	Sweets^c^	Sweets in general (desserts)
		Frosted cake
		Chocolate
	Fatty/processed meats^b^	Fried chicken
		Sausage
		Wiener
		Pork rinds
		Ham/salami
	Fats^b^	Margarine
		Butter
		Lard

*Source: Adapted from Ministério da Saúde do Brasil (Vigitel 2016) (28)*.

a *Healthy eating indicators, if they were consumed five times/week or more. For the group of “other vegetables,” yam, cassava, and potatoes were excluded*.

b *Unhealthy eating indicators, if they were consumed at least once a week*.

c *Unhealthy eating indicators, if they were consumed five times/week or more*.

d *A kind of cheese very popular in the state of Minas Gerais, Brazil*.

e *Traditional Brazilian food prepared with wheat flour dough and various fillings*.

### Statistical Analysis

The profile of food consumption was obtained from categorizing the individuals based on the frequency of consumption of the food groups listed in the FFQ. To obtain this variable, we used the two-step cluster analysis (TSC), which is a consistent method used for grouping the subjects and aggregate units based on the characteristics that they possess (29). In the cluster analysis, the idea is to explore the homogeneity in each cluster and the heterogeneity among the clusters to define a data structure. When the probability of the presence of a given characteristic in a cluster is above 50%, this characteristic determines the cluster format (29). In this study, the main characteristic was the categorical variables of the regular consumption of each food group, that is, the presence or absence of food groups. The unit of analysis was the individual, and in the pre-agglomeration step, TSC checked each data; in this case, each senior pointing out whether the individual could be added to a previously formed cluster or a new cluster needed to be created for him/her (30).

The descriptive analysis includes the estimates of medians and interquartile ranges for the quantitative variables and the frequency distribution for the categorical variables. Multinomial logistic regression was used to identify the association between the independent variables of interest in this study and the dependent variable, the clusters of food consumption. Crude and adjusted odds ratios (OR) were estimated as well as their 95% confidence intervals (CI). In this study, the independent variables that were associated with the dependent variable with *p* < 0.20 in the univariate stage were included in the multiple multinomial logistic regression model according to Hosmer and Lemeshow (31). Data analysis was performed using the IBM SPSS software, version 20.0. The level of significance for rejecting the null hypothesis was α = 0.05.

## Results

Regarding the socioeconomic characteristics of the participants, the majority were female, between 60 and 69 years old, had <8 years of study, and resided with someone else (Table 2). Regarding income, most seniors fell under the second quartile (individual income between US$ 211.00 and US$ 237.49 per month). The lower limit corresponded to the minimum wage converted into American dollars according to the rates at the time of the study. Most of the seniors perceived their health to be regular and had a history of one to five doctor's appointments, with 15.1% reporting a history of hospitalization in the year preceding the survey. Functional ability was considered inadequate for about 16% of the individuals, and a minority reported difficulty with eating, difficulty with food preparation, and some decrease in food intake over the 3 months prior to the survey. Polypharmacy was identified in a third of the participants as well as the consumption of alcoholic beverages; however, the majority had never smoked. Regarding the diagnosis of non-communicable diseases, high blood pressure was the most prevalent, followed by a history of dyslipidemia, a history of diabetes, and depression. Anthropometric variables are presented as median and interquartile range (Table 2).

**Table 2:** Socioeconomic characteristics, health and use of health services, lifestyle, and anthropometric indicators in the older adult sample, Viçosa, State of Minas Gerais, Brazil, 2009 (*n* = 621).

**Variable**	***n***	**%**	**Median**	**Interquartile range**
**Sex female**	331	53.3		
Age range				
60–69	311	50.1		
70–79	216	34.8		
≥80	94	15.1		
Education level				
Never studied	95	15.3		
Less than eight years of study	397	63.9		
Eight years of study or more	129	20.8		
Individual income/month (quartiles)				
Q1 (US$ 0–210.99)	72	11.6		
Q2 (US$ 211.00–273.49)	234	37.7		
Q3 (US$ 237.50–670.99)	153	24.6		
Q4 (≥ US$ 671.00)	153	24.6		
Lived alone	66	10.6		
Self-perception of health^a^				
Very good/good	272	43.8		
Regular	289	46.5		
Poor/very poor	38	6.1		
Number of doctor's appointments (in the preceding year)^a^				
None	45	7.2		
1–5	449	72.3		
≥6	126	20.3		
**History of at least one hospitalization (in the preceding year)**	94	15.1		
**Functional disability**	100	16.1		
**Difficulty with eating**	53	8.5		
**Difficulty with food preparation**	66	10.6		
**Polypharmacy (in the preceding 15 days)**	224	36.1		
**History of diabetes**	139	22.4		
**History of dyslipidemia**	353	56.8		
**History of high blood pressure**	475	76.5		
**History of depression**	117	18.8		
Consumption of alcoholic beverages^a^				
Yes	209	33.7		
No, but used to drink formerly	205	33.0		
Never drank at all	206	33.2		
Smoking habit^a^				
Yes	67	10.8		
No, but used to smoke formerly	207	33.3		
Never smoked at all	345	55.6		
Decrease in the food intake (in the preceding 3 months)	78	12.6		
Anthropometric variables^b^				
BMI (kg/m^2^)			26.37	23.49–29.53
WC (cm)			95.30	87.60–103.10
WHR			0.96	0.91–1.00
WHtR			0.60	0.55–0.65

*BMI, body mass index; WC, waist circumference; WHR, waist-to-hip ratio; WHtR, waist-to-height ratio*.

a *Not informed by a part of the sample*.

b *Not measured/calculated in a part of the sample*.

Most of the individuals used to consume foods that were listed under “healthy eating indicators,” except for skim milk and low-fat dairy products. Of note is the regular consumption (five times or more per week) of beans by nearly the entire sample. In relation to “unhealthy eating indicators,” we identified a low frequency of consumption of sweets and soda. On the other hand, the consumption of fatty/processed meat at least once a week was reported by the majority of the seniors (Table 3).

**Table 3:** Frequency of regular consumption of food groups, listed under healthy and unhealthy eating indicators, Viçosa, State of Minas Gerais, Brazil, 2009 (*n* = 621).

**Food groups**	**Consumption**	
	***n***	**%**
**Healthy eating indicators**		
Beans	581	93.6
Vegetables	443	71.3
Green vegetables	440	70.9
Fruits	443	71.3
Skim milk and low-fat dairy products	170	27.4
**Unhealthy eating indicators**		
Fatty/processed meat	553	89.0
Fat	385	62.0
Whole milk	359	57.8
Processed foods in general	183	29.5
Sweets	54	8.7
Soda	13	2.1

**Figure 1:**
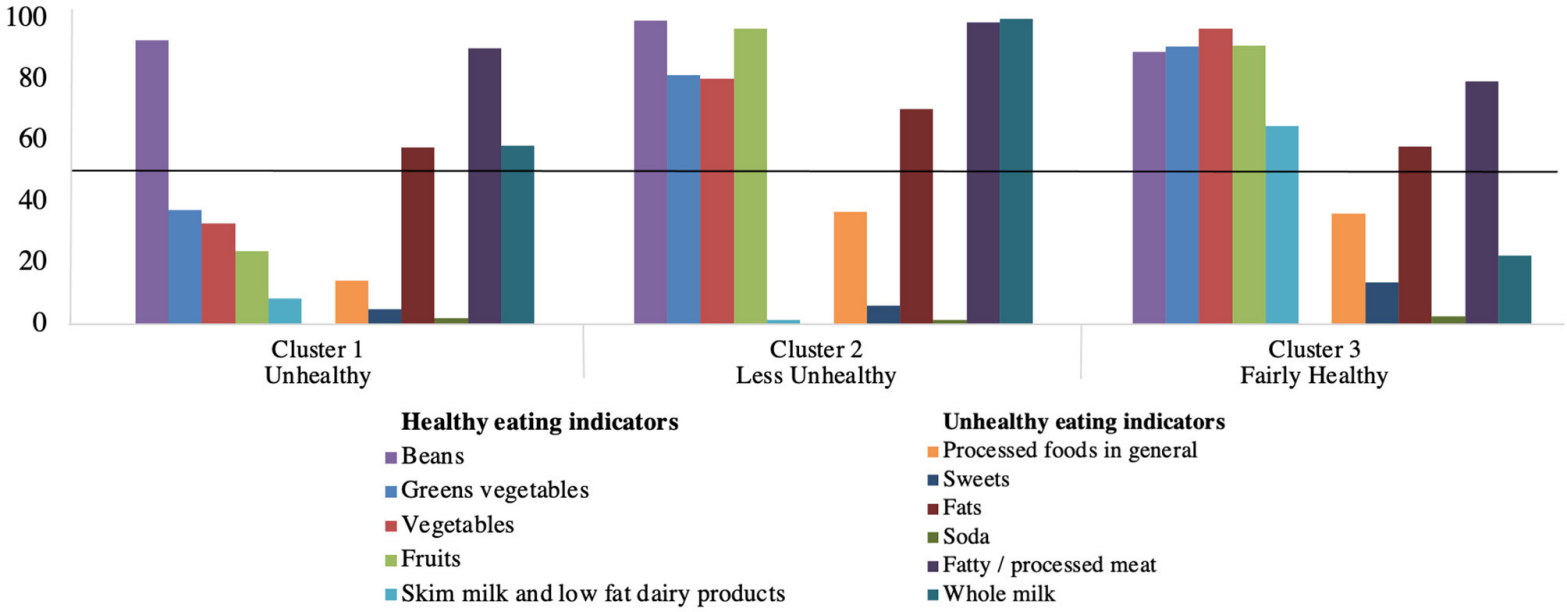
Consumption frequency of the groups of healthy and unhealthy eating indicators, according to the clusters of older adults, Viçosa, State of Minas Gerais, Brazil, 2009 (*n* = 621). When the probability of regular consumption of an indicator is above 50% (y-axis), this food group is a characteristic that determines the cluster format.

The analysis generated three clusters using the information regarding the individual's food consumption, which were named according to the behavior of the seniors that they included. The first was “unhealthy,” the second “less unhealthy,” and the third “fairly healthy” (Figure 1). The cluster “unhealthy” (32.2% of the sample) was characterized by older adults who regularly consumed beans, fats, fatty/processed meats, and whole milk. The cluster “less unhealthy” was formed by 30.6% of the participants who regularly consumed beans, green vegetables, vegetables, and fruits as well as consumed fats, fatty/processed meats, and whole milk. The cluster “fairly healthy” was represented by 37.2% of the individuals who regularly consumed beans, green vegetables, vegetables, fruits, fats, fatty/processed meats, skim milk, and low-fat dairy products. Among the healthy eating indicators, we highlight the consumption of beans, as in the three clusters almost all the participants consumed this food regularly (five or more times per week). Similar behavior was observed in relation to the consumption of fats and fatty/processed meats, characterizing them as unhealthy eating indicators that were present in all clusters. Some food groups had a low frequency of consumption in all clusters, such as processed foods in general, sweets, and soda. Processed foods in general had an even lower consumption frequency in cluster 1, and the sweets were slightly more frequent in cluster 3.

In the univariate analysis, the variables associated with the clusters “unhealthy” and “less unhealthy” were education level, individual income, number of doctor's appointments, history of diabetes, history of dislipidemia, polypharmacy, consumption of alcoolic beverages, smoking habbit, reduction in food ingestion, and waist circumference (data not shown). The OR and the respective confidence intervals (95% CI) of the multiple analyses between the socioeconomic and health conditions and clusters of food consumption in this older adult population are presented in Table 4. Individuals who never studied or with <8 years of study and those who reported at least six doctor's appointment in the prior year were more likely to belong to the clusters “unhealthy” and “less unhealthy.” The seniors in quartiles 1 and 2 of individual income, those who reported one to five doctor's appointment in the prior year, and the former smokers had a greater chance to belong to the cluster “unhealthy.” There was a borderline association of the cluster “unhealthy” with a history of diabetes (OR 0.6; 95% CI 0.3,1.0; *p* = 0.051), and there was also a borderline association of the cluster “less unhealthy” with a history of dyslipidemia (OR 0.6; 95% CI 0.4,1.0; *p* = 0.054).

**Table 4:** Final results of the multiple analysis of the association among the sociodemographic and health conditions and the food consumption clusters^a^, Viçosa, State of Minas Gerais, Brazil, 2009 (*n* = 621).

**Variable**	**Cluster 1**	**Cluster 2**
	**Unhealthy**	**Less unhealthy**
	**OR (CI 95%)**	**OR (CI 95%)**
**Education level**		
Eight years of study or more	1.0	1.0
Less than 8 years of study	**3.8 (1.9–7.5)**	**2.8 (1.6–4.9)**
Never studied	**10.7 (4.2–27.7)**	**5.4 (2.2–13.2)**
**Individual income/month (quartiles)**		
Q4 (≥US$ 671.00)	1.0	1.0
Q3 (US$ 237.50–670.99)	1.5 (0.8–3.1)	1.1 (0.6–2.0)
Q2 (US$ 211.00–273.49)	**2.3 (1.2–4.6)**	1.1 (0.6–2.0)
Q1 (US$ 0–210.99)	**2.4 (1.1–5.5)**	1.1 (0.5–2.3)
**Number of doctor's appointments**		
None	1.0	1.0
1–5	**0.3 (0.1–0.8)**	0.6 (0.2–1.6)
≥6	**0.3 (0.1–0.9)**	**0.3 (0.1–0.9)**
**History of diabetes**		
No	1.0	1.0
Yes	0.6 (0.3–1.0)	0.8 (0.4–1.3)
**History of dyslipidemia**		
No	1.0	1.0
Yes	0.7 (0.5–1.2)	0.6 (0.4–1.0)
**Polypharmacy**		
No	1.0	1.0
Yes	0.7 (0.4–1.1)	0.8 (0.5–1.2)
**Consumption of alcoholic beverages**		
Never drank at all	1.0	1.0
No, but used to drink formerly	1.4 (0.8–2.5)	1.1 (0.6–1.9)
Yes	0.8 (0.4–1.5)	0.9 (0.5–1.6)
**Smoking habit**		
Never smoked at all	1.0	1.0
No, but used to smoke formerly	**1.8 (1.1–3.1)**	1.6 (0.9–2.6)
Yes	2.0 (0.9–4.2)	0.9 (0.4–1.9)
**Reduction in food ingestion**		
No	1.0	1.0
Yes	1.6 (0.8–3.4)	1.7 (0.8–3.5)
**WC (median** **=** **95.3cm)**		
Lesser than the median	1.0	1.0
Larger than or equal to the median	0.9 (0.9–1.0)	1.0 (0.9–1.0)

a *For this analysis, the cluster “fairly healthy” was the reference category*.

*CI95%, 95% confidence interval; OR, odds ratio; WC, waist circumference*.

*Values in bold indicate factors independently associated with the “unhealthy” and “less unhealthy” clusters*.

## Discussion

This is the first Brazilian population-based study that obtained and described three profiles of older adults from the two-step cluster analysis concerning food consumption. We identified the food groups and eating habits that contributed to characterize these profiles, and we estimated the association of social and demographic variables, health and use of health services, lifestyle, and anthropometric indicators with these profiles.

The first cluster was identified as “unhealthy.” Despite being characterized by the regular consumption of beans, a healthy eating indicator, their other three food groups were unhealthy eating indicators (whole milk, fatty/processed meats, and fats). Moreover, most of the seniors in the cluster “unhealthy” were not used to consuming fruits and vegetables regularly. The second cluster was classified as “less unhealthy” because, although it was characterized by the presence of fruits, green vegetables, and beans, it also included whole milk, fatty/processed meats, and fats. The third cluster was classified as “fairly healthy” because it differed from cluster 2, as it included skim milk and low-fat dairy products, and from cluster 1 by the consumption of all healthy eating indicators. Thus, this cluster represents individuals with a healthier food profile, as compared to the other two clusters, but is not completely healthy because it was also characterized by the consumption of fats and fatty/processed meats.

Changes in food consumption caused by modernization are of concern in all population groups, as they are characterized by the replacement of fresh foods rich in nutrients with processed foods rich in sugar, fat, and additives (32). When it turns to older adults, monotonous food choices with low nutritional quality are the greatest risk (33, 34). Nevertheless, in this study, we observed a mix of high consumption of healthy eating indicators, such as fruits and vegetables, and unhealthy eating indicators, such as whole milk, fatty/processed meats, and fats. The consumption of processed foods, sweets, and soda did not characterize any cluster, which shows that the older adults in this sample still remained on a traditional diet despite that the cluster “unhealthy” had shown little variety.

Lower education level was found to be directly associated with the clusters “unhealthy” and “less unhealthy.” Low education level is often a factor that negatively impacts the eating habits and leads to a greater risk of malnutrition as reported by previous epidemiological studies (3, 16, 17, 32, 35, 36). Giuli et al. (3), in an Italian study, identified that seniors with a higher education level consumed more beans, cereals, fruits, fish, red meat, and dairy products. In Brazil, another study observed a lower education level among aged individuals whose families revealed food insecurity (35), and Gadenz and Benvegnú (13) identified a positive association between more years of study and the consumption of fruits and vegetables by aged individuals. In the present study, the cluster “unhealthy” is an example of worse nutrition associated with low education. With more years of study, there may be greater access to information about the quality of food and the contribution of each food group to health. In addition, more often, higher education is also related to higher income, which will be discussed later. The only difference between the clusters “fairly healthy” and “less unhealthy” was the regular consumption of skim milk and low-fat dairy products in the first category and the consumption of whole milk in the second. Considering this, the results of the current study suggest that a higher education level (8 years of study or more) promotes access to information on healthy eating, making it reasonable to assume that it influences the habit of replacing whole milk with skim products when necessary. A review from Netherlands about dietary guidances confirms that dairy products are part of these food-based dietary guidelines because of their nutrient richness and emphasizes that low-fat or skimmed versions must be generally advocated (37). This result may also indicate that nutrition will be better with the tendency of increase in the education of older adults, a hypothesis that requires further investigation in the future. For now, this result evidences the need to promote nutrition education suitable for the low-educated older adult population with appropriate communication.

Lower income was also found to be directly associated with the cluster “unhealthy,” that is, the seniors included in this cluster were more likely to possess an individual income less than US$ 237.50 per month, marginally higher than the minimum wage of the time, when compared with the cluster “fairly healthy.” Assumpção and colleagues also reported results in which seniors earning higher incomes revealed higher healthy eating scores (15). Total household income was significantly and positively associated with fruits and vegetables intake among older adults in the Canadian study of Riediger and Moghadasian (32). Despite the great offer and diversity of fruits and vegetables in Brazil, even at lower costs, it is still expected that the aged individuals with higher personal incomes enjoy greater purchasing power, which may encourage food choices with more quality and variety. The association observed between the lower socioeconomic level and low quality of diet suggests unequal access to food among the older adults, which is supported by the evidence that the cluster “unhealthy” was characterized by people with a more monotonous food consumption, lacking the important variety and quality provided by fruits, vegetables, skim milk, and low-fat dairy products. In this sense, policies must be developed to reduce income inequality and guarantee access to adequate food for older adult populations.

The variable “number of doctor's appointments” was inversely associated with the clusters “unhealthy” and “less unhealthy.” The individuals assigned to the cluster “fairly healthy” had a higher number of consultations in the year preceding the survey. Gadenz and Benvegnú identified a similar behavior with aged individuals, revealing the direct association between adequate diet and a history of consultation with a nutritionist (13). An inference that can be drawn is that, because these seniors had sought more healthcare, they enjoyed greater access to information related to wise eating choices and therefore consumed more food considered as healthy eating indicators, such as fruits, vegetables, skim milk, and low-fat dairy products.

From the univariate analysis, the history of diabetes was inversely associated with the cluster “unhealthy,” and the history of dyslipidemia was inversely associated with both clusters “unhealthy” and “less unhealthy.” Both did not remain in the final model, but they were in the significance threshold (*p* = 0.051 for diabetes and *p* = 0.054 for dyslipidemia). It is possible that these morbidities resulted in greater demand/utilization of health services by the seniors and, consequently, provided greater access to information, culminating in better eating habits. This possible behavioral change, called reverse causality, was also reported by Assumpção and colleagues, who observed a better quality of diet among older adults with a reported diagnosis of diabetes (15). Thus, it is possible that the best diet quality can be due to the diagnosis of non-communicable chronic diseases, which demands the adoption of a healthy diet for accurate treatment.

Another condition directly associated with the cluster “unhealthy” was having been a former smoker. The individuals in this profile were more likely to have smoked and stopped this habit at some point in their lives. Due to the lack of information regarding the time of smoking exposure and the time that had lapsed between the interview and smoking cessation, this data can be extremely variable, and the relationship between this old habit and the present diet is not precise. Smoking is known to change the palatability of some foods, such as fruits, vegetables, dairy products, and even water (38), and the cessation of this habit can reawaken the ability to experience the taste of these foods (38, 39). Besides that, the adoption of a healthier behavior, such as smoking cessation, should be accompanied by an improvement in other aspects, like eating habits. Perhaps these changes are motivated by different reasons, which explains the results observed. The association between the current smoking habit and the cluster “unhealthy” in multiple analysis showed a trend toward significance (*p* = 0.066). Studies show the association between smoking and poor eating habits (15, 38, 40, 41). It is possible that the small number of smokers in this study (*n* = 67; 10.8%) has restricted the ability to identify this association in the sample under study.

It is worth emphasizing that all methods of evaluation of food intake have limitations, such as the possibility of memory bias, especially in aged individuals. The FFQ is considered an efficient tool for identifying the usual food consumption, speccially when conducted by trained interviewers, besides having a low cost and simple application (42). In addition, we believe that the monotony of the diet of the Brazilian older adults (33, 34) minimizes the limitations of the FFQ and the possible distortions in the results of the food profile of this population. The qualitative characteristic of the instrument used has not enabled the evaluation of the food portions consumed by the seniors. This fact impairs the comparison with some recommendations and a deeper evaluation of the diet.

A limitation inherent in the cross-sectional design is the inability to establish the temporal relationship of some of the associations observed. Nevertheless, they are consistent with the literature and generate hypotheses that need to be explored in longitudinal studies in order to recognize the determinants of the eating habits of older adults, and despite the limitations, they are important as a nutritional advice for these invididuals.

A possible limitation of the study is the fact that the health conditions and drug utilization information are self-reported. However, the way to obtain this information was based on instruments adopted by large national surveys that have already reported its satisfactory validity.

In conclusion, we identified three clusters called “unhealthy,” “less unhealthy,” and “fairly healthy” based on data from the food consumption of older adults in Brazil. Although all clusters have been characterized by the consumption of some unhealthy foods, the subjects presented traditional eating habits in this study, especially showing a low consumption of soda, sweets, and processed foods in general and a high consumption of beans. However, the cluster “unhealthy” was composed of individuals without the habit of regular consumption of fruits and vegetables. We observed an association between low education level, low income, few doctor's appointments, and the fact of being a former smoker with poor diet quality. The evidence indicates the profile of seniors who require greater attention and care related to improved nutrition. Nutrition education public policies and health actions must be focused on and appropriate for the illiterate or semi-literate aged individuals with lower income and those who do not seek guidance. In light of the obtained results, efforts should be made to encourage older adults to keep the traditional and healthy eating habits, with greater access to consumption of fruits, vegetables, and beans and low consumption of soda, sweets, and processed foods.

## Data Availability Statement

The raw data supporting the conclusions of this article will be made available by the authors, without undue reservation.

## Ethics Statement

This study was reviewed and approved by Research Ethics Committee of the Federal University of Viçosa (ref. 27/2008). The participants provided their written informed consent to participate in this study.

## Author Contributions

LL, MP, and AR contributed to the conception and design of the study. AR contributed to the acquisition of data. LL and AR wrote the first draft with contributions from MP, LA, and CM. All the authors reviewed and commented on the subsequent drafts of the manuscript.

## Conflict of Interest

The authors declare that the research was conducted in the absence of any commercial or financial relationships that could be construed as a potential conflict of interest.
